# Age-Related Differences in Ocular and Cardiovascular Responses and Recovery After Graded Motor–Cognitive Physical Activity

**DOI:** 10.3390/healthcare14101290

**Published:** 2026-05-09

**Authors:** Teresa Zwierko, María Dolores Morenas-Aguilar, Wojciech Lubiński, Krystian Panek, Sonja Himmel, Thorben Hülsdünker, Jesús Vera

**Affiliations:** 1 Institute of Physical Culture Sciences, Center for Structural and Functional Human Research, University of Szczecin, 71-065 Szczecin, Poland; 2 Department of Physical Education and Sport, Faculty of Sport Sciences, University of Granada, 18071 Granada, Spain; mdmorenas@ugr.es; 3 The Second Department of Ophthalmology, Pomeranian Medical University, 70-111 Szczecin, Poland; lubinski@pro.onet.pl; 4 Department of Physical Culture and Health, Student of Sport Diagnostics Faculty, University of Szczecin, 71-065 Szczecin, Poland; 228355@stud.usz.edu.pl; 5 Doctoral School, University of Szczecin, 70-384 Szczecin, Poland; 6 HAAK Therapy Centre, 61-782 Poznań, Poland; psycholog.himmel@gmail.com; 7 Department of Sport, LUNEX, 4671 Differdange, Luxembourg; thuelsduenker@lunex.lu; 8 Luxembourg Health & Sport Science Research Institute, 4671 Differdange, Luxembourg; 9 CLARO (Clinical and Laboratory Applications of Research in Optometry) Research Group, Department of Optics, University of Granada, 18071 Granada, Spain; veraj@ugr.es; 10 New England College of Optometry, Boston, MA 02115, USA

**Keywords:** aging, dual-task, intraocular pressure, ocular perfusion pressure, blood pressure, heart rate

## Abstract

**Highlights:**

**What are the main findings?**

- Increasing motor–cognitive task complexity progressively changed ocular and cardiovascular responses.
- Age-related differences were most evident in task performance, higher relative heart rate, and intraocular pressure during early recovery.

**What are the implications of the main findings?**

- Combined ocular and cardiovascular measures may help characterize physiological adaptation to motor–cognitive load.
- Task progression and early recovery should be considered when designing motor–cognitive exercise protocols for adults across age groups.

**Abstract:**

**Background/Objectives:** This study examined age-related differences in ocular and cardiovascular responses to graded motor–cognitive task complexity in young and older adults. **Methods:** Forty-three healthy adults participated, including 21 young adults (27.1 ± 7.7 years) and 22 older adults (63.1 ± 7.8 years). Participants completed three tasks of progressively increasing motor–cognitive complexity. Intraocular pressure (IOP), ocular perfusion pressure (OPP), blood pressure, and heart rate were measured at baseline, after each task block, and at a post-task recovery assessment. **Results:** Significant condition effects were found for IOP (*p* = 0.012, η^2^p = 0.08), OPP (*p* < 0.001, η^2^p = 0.76), systolic blood pressure (*p* < 0.001, η^2^p = 0.51), and relative heart rate (*p* < 0.001, η^2^p = 0.40). Condition × group interactions were significant for IOP (*p* = 0.004, η^2^p = 0.09), OPP (*p* = 0.002, η^2^p = 0.10), and systolic blood pressure (*p* = 0.003, η^2^p = 0.11). Post hoc analyses showed that young adults exhibited a decrease in IOP from the first task block to recovery (*p* = 0.011, *d* = 0.35), whereas older adults showed a return toward baseline values. Relative heart rate was consistently higher in older adults across the protocol (*p* < 0.001, η^2^p = 0.29). **Conclusions:** Increasing motor–cognitive task complexity elicited progressive ocular and cardiovascular responses. The findings suggest that aging may influence the pattern of physiological adaptation to graded motor–cognitive load, particularly during early recovery. Concurrent assessment of ocular and cardiovascular measures may help characterize these response patterns.

## 1. Introduction

Cognitive functions such as working memory, cognitive flexibility, decision-making, and visuospatial perception support safe and effective performance when rapid information processing must be paired with precise motor responses. This is particularly relevant in dynamic, time-pressured settings, such as sports and many occupational tasks [1,2], and it also contributes to maintaining everyday functioning with age [3]. When cognitive demands are high, mental fatigue may accumulate and, combined with physical effort, can degrade performance under sustained loads [4,5]. Although regular physical activity benefits cognitive health, age-related changes in executive functions and perceptual–motor coordination may still challenge functional capacity [6]. Therefore, motor–cognitive approaches are increasingly viewed as a way to support functioning and well-being in older adults [7].

Motor–cognitive tasks are relevant not only from the perspective of performance but also because they are associated with measurable physiological responses. Cognitive load can affect cardiovascular dynamics and autonomic regulation [8] and may also influence physiological adjustment during exercise [9]. In everyday settings, motor actions are rarely performed in isolation. They typically require continuous monitoring of sensory information and ongoing response selection. In this context, dual-task paradigms provide a useful framework for studying physiological responses under conditions that more closely resemble real-world demands, particularly in relation to healthy aging [10]. As motor–cognitive demands increase, they may place greater demands on attentional control and working memory. This is especially relevant in midlife and older age, when cognitive function contributes substantially to performance in combined cognitive and locomotor tasks [11]. Psychophysiological accounts of effort mobilization suggest that physiological activation tends to increase with task difficulty as long as task success remains attainable [12]. Together, these considerations provide a rationale for examining whether graded increases in motor–cognitive task complexity are accompanied by progressively stronger physiological responses.

These relationships are also likely to vary with age. Research on resource engagement has shown that the association between task difficulty, perceived attainability, goal value, and cardiovascular responses may differ between younger and older adults [13]. Systolic blood pressure has been proposed as a useful indicator of task engagement and as a marker of age-related differences in resource mobilization during task performance [14]. This suggests that aging may influence both the magnitude of physiological responses during demanding tasks and the physiological status observed after task completion.

In addition to cardiovascular indices, ocular measures may also provide valuable insights in health contexts. IOP and OPP are relevant for ocular homeostasis. Importantly, OPP integrates systemic circulation through arterial blood pressure with an ocular component through IOP, making it a physiologically meaningful link between cardiovascular activation during effort and ocular status. Available evidence indicates that the direction of IOP changes depends on the type and conditions of the load. Aerobic exercise is typically associated with a transient decrease in IOP [15,16]. In contrast, resistance and isometric exercise more often produce temporary increases, with reviews highlighting breathing patterns and body position as key modifiers of this response [17]. In addition, psychological factors such as anxiety and emotional strain may also contribute to increases in IOP [18]. These considerations are primarily relevant to ocular physiology and may also provide valuable insights in health-related contexts [19].

Because motor and cognitive demands often co-occur, it is justified to examine IOP and OPP under graded motor–cognitive load. However, evidence remains limited regarding the simultaneous assessment of IOP, OPP, and cardiovascular responses during graded motor–cognitive protocols, particularly with attention to age-related differences. The aim of the present study was to evaluate age-related differences in ocular and cardiovascular responses to increasing levels of motor–cognitive task complexity. Using a graded dual-task protocol, we assessed IOP, OPP, and cardiovascular indices at rest, during task blocks of increasing difficulty, and at a post-task recovery assessment. We expected that increasing task complexity would be associated with changes in IOP and with stronger cardiovascular responses, consistent with psychophysiological accounts of effort mobilization [12] and with previous evidence that psychological strain can induce short-term changes in IOP [18]. We further hypothesized that these physiological responses would differ by age. In particular, older adults were expected to show a different response profile and a less complete return toward baseline at recovery, consistent with previous work on age-related differences in resource engagement and cardiovascular responding during task performance [13,14].

## 2. Materials and Methods

### 2.1. Participants

In this study, we aimed to recruit healthy community-dwelling adults in two age groups, young adults and older adults, in an approximately 1:1 ratio to examine age-related differences in physiological regulation. All participants provided written informed consent after receiving a comprehensive explanation of the study objectives, procedures, and potential risks.

A total of 64 individuals were initially screened. Of these, 21 were excluded for reasons including lack of interest in participation, failure to meet inclusion criteria, or physical limitations that could interfere with safe task execution. During recruitment, participants were asked about their habitual physical activity as part of the general screening procedure; however, this variable was not formally assessed or included in the analyses. The final sample included 43 healthy participants with baseline IOP values ranging from 11 to 21 mmHg. Participants were allocated to two groups based on age: the young adult group comprised 21 individuals (11 men), and the older adult group comprised 22 individuals (9 men).

Based on a previously reported effect size (*f* = 0.33) for IOP changes in response to cognitive load in healthy individuals [18], the present sample of 43 participants yielded a statistical power of 0.95 for detecting within-subject effects in repeated-measures ANOVA (α = 0.05).

Young adults had a mean age of 27.14 years (SD = 7.65; range: 20–42), a mean height of 174.38 cm (SD = 11.66; range: 160–206), and a mean body mass of 73.14 kg (SD = 15.50; range: 53–120). Older adults had a mean age of 63.14 years (SD = 7.75; range: 51–76), a mean height of 169.41 cm (SD = 11.27; range: 153–186), and a mean body mass of 72.23 kg (SD = 14.34; range: 51–112).

Inclusion criteria required the absence of systemic or neurological conditions that could affect visual function or visuospatial processing, including diabetes with ocular complications, hypertensive retinopathy, multiple sclerosis, or a history of stroke. All participants had corrected distance visual acuity to ensure standardized visual conditions during task execution.

Detailed participant characteristics are presented in Table 1.

### 2.2. Procedure and Experimental Protocol

Participants completed the experiment over two test days, each lasting approximately 30 min. All tasks were performed using SKILLCOURT technology (SKILLCOURT GmbH, Bergrheinfeld, Germany) on a dedicated 4 × 4 m field.

**Figure 1:**
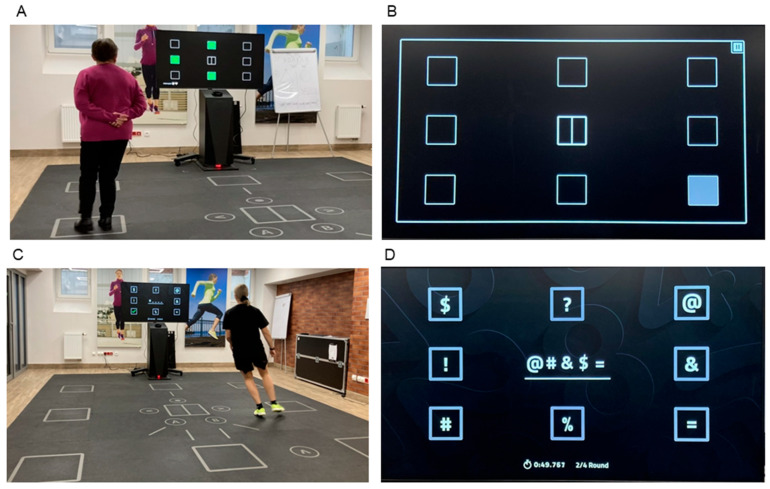
SKILLCOURT setup in the laboratory during the visual–spatial memory span test, forward version (**A**), with a participant standing on the designated field. Example from the visual–spatial memory span test showing a single glowing square on the screen as part of the sequence (**B**). SKILLCOURT setup during the motor–cognitive exercise session (**C**), with a participant actively moving on the field during block 3. Illustration of the motor–cognitive memory task with five symbols (block 3) displayed on the screen (**D**).

On the first day, participants completed a motor–cognitive visual–spatial memory span test, including both forward (VSMS-F) and backward (VSMS-B) versions, designed to assess the capacity to store and manipulate visuospatial information in working memory. This task, based on the Corsi block-tapping test [20], evaluates the ability to encode, retain, and recall visual stimuli presented in a specific spatial configuration while integrating movement (Figure 1A,B). The sequence started with a small number of items (two) and gradually increased as the test progressed, up to a maximum of eight items. Each square appeared individually for one second before the next one was presented. After the full sequence was displayed, the squares disappeared from the screen, and participants were instructed to replicate the sequence by physically moving across the field and selecting the corresponding squares in the same order. In the second phase of the task, participants performed the sequences in reverse order (backward). The test continued until the participant failed to correctly reproduce the sequence in two consecutive attempts at the same sequence length. The longest correctly reproduced sequence determined the participant’s visual memory span. Prior to testing, a familiarization trial was conducted to ensure that participants understood the procedure.

On the second day, participants completed a motor–cognitive exercise session consisting of three tasks of increasing task complexity. In each task, participants were shown a sequence of symbols presented at the center of the screen and were required to memorize their order. Then, they had to replicate the sequence by navigating to the corresponding target squares on the field, each permanently associated with a specific symbol (Figure 1C). Each trial began with the participant positioned at the central square of the field, after which the symbols disappeared. Participants were instructed to move as quickly as possible to each square corresponding to the memorized sequence of symbols.

The first task (block 1), involving three symbols, included six trials. The second task (block 2), using four symbols, consisted of five trials. The third and most challenging task (block 3), which involved five symbols (Figure 1D), was completed in four trials. The total completion time for each task was recorded in minutes. The three task blocks were performed in a fixed order from lower to higher difficulty to preserve the graded structure of the protocol and minimize potential carryover effects related to fatigue or prior exposure to more demanding conditions, consistent with previous SKILLCOURT-based research [21]. Within each block, the number of symbols was predefined by the protocol, whereas the symbol sequences presented in individual trials were generated in random order. Five-minute breaks were provided between tasks to help maintain consistent task execution across blocks while preserving the progressive nature of the protocol.

### 2.3. Physiological and Subjective Outcome Measures

IOP, blood pressure (BP), and heart rate (HR) were assessed to evaluate physiological responses to graded motor–cognitive task complexity. All physiological measurements were conducted on the second testing day during the motor–cognitive exercise session, following a standardized protocol. Measurements were obtained at baseline (pre-task), immediately after each task block (blocks 1–3), and during recovery (five minutes after completion of the final block), yielding five assessment points per participant.

IOP was assessed in the right eye using rebound tonometry (iCare 200, Icare, Tiolat Oy, Helsinki, Finland), a method widely used in experimental and applied settings due to its rapid acquisition, noninvasiveness, and high participant tolerance [22,23,24]. Participants were seated and instructed to maintain an upright head position and fixate on a distant target to minimize accommodation-related effects. At each assessment point, six consecutive readings were automatically obtained by the device on the central cornea, and the mean IOP value was recorded. The device provides an integrated quality indicator reflecting within-measurement variability. Only measurements classified as acceptable according to the manufacturer’s predefined criteria were retained. When excessive variability was detected, most commonly due to blinking, eye movements, unstable fixation, or suboptimal probe alignment, the measurement was repeated. All IOP assessments were performed by the same trained examiner to ensure procedural consistency.

BP was measured using a clinically validated wrist-worn digital automatic monitor (RX3, Omron, Hoofddorp, The Netherlands). To enhance measurement reliability, two consecutive BP readings were obtained at each time point, and the mean value was used for further analyses. All measurements were performed under standardized conditions, with participants seated and the wrist supported at heart level throughout the procedure. Given that wrist-based measurements are particularly sensitive to positioning error, strict attention was paid to correct wrist placement during each assessment. BP values were used to calculate OPP, which was determined using the following formula: OPP = (95/140 × MAP) − IOP, where mean arterial pressure (MAP) was defined as diastolic blood pressure (DBP) + 1/3 (systolic blood pressure (SBP) − DBP) [25]. Additionally, blood pulse pressure (BPP) was calculated as the difference between SBP and DBP.

Heart rate (HR) was measured at baseline, immediately after each task block, and during recovery using the same automatic BP monitor (RX3, Omron, Hoofddorp, The Netherlands), which derives HR values from pulse detection during cuff inflation/deflation. For analysis, relative HR was expressed as a percentage of estimated maximal HR, calculated using the formula 220 − age [26], to account for interindividual and age-related differences in cardiovascular capacity. This approach was used to provide a more comparable index of cardiovascular effort across the two age groups, as the same absolute HR may represent a different physiological load in young and older adults.

All physiological data were visually inspected prior to analysis to identify potential artifacts or implausible values (e.g., abrupt discontinuities, values outside physiological ranges, or inconsistent patterns across consecutive measurements). Any measurement affected by technical error or participant movement was immediately repeated according to the standardized protocol, and only acceptable readings were retained for analysis. As a result, no missing values remained in the final dataset.

**Figure 2:**
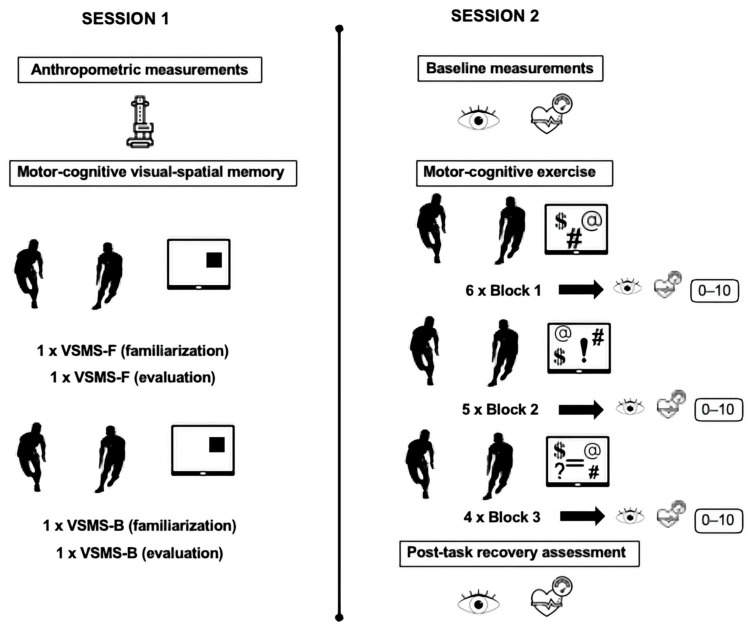
Schematic overview of the study design and experimental protocol. VSMS-F = visual–spatial memory span test, forward version; VSMS-B = visual–spatial memory span test, backward version.

Following completion of each task block (blocks 1–3), participants rated the perceived task complexity of the task using a numerical rating scale ranging from 0 (“very easy”) to 10 (“extremely difficult”). This measure provided a subjective index of perceived complexity across task blocks. A schematic overview of the study design and experimental protocol is shown in Figure 2.

### 2.4. Statistical Analysis

Descriptive data are presented as means ± standard deviation. The normal distribution of the data was assessed using the Shapiro–Wilk test, and the homogeneity of variances with the Levene’s test (*p* > 0.05). Between-group comparisons of baseline values for the dependent variables were conducted using independent samples *t*-tests for normally distributed data and the Mann–Whitney U test for non-normally distributed variables. Perceived task complexity ratings recorded after each of three motor–cognitive task blocks were analyzed using a repeated-measures analysis of variance (ANOVA), with “task complexity” (block 1, block 2 and block 3) as the within-participants factor and “group” (young adults vs. older adults) as the between-participants factor.

For the remaining physiological dependent variables (i.e., IOP, SBP, DBP, MAP, OPP, and RHR), separate 5 × 2 repeated-measures ANOVAs were performed, with condition (baseline, block 1, block 2, block 3, and recovery) as the within-participants factor and group (young adults vs. older adults) as the between-participants factor, to evaluate task-related changes across the motor–cognitive protocol in both groups.

We reported Cohen’s d and partial eta-squared (η^2^p) as effect size indices. For interpretation, values of 0.2, 0.5, and 0.8 were considered small, medium, and large effects, respectively, for Cohen’s *d*; and values of 0.01, 0.06, and 0.14 were interpreted as small, medium, and large effects, respectively, for η^2^p [27]. Post hoc comparisons were corrected using the Holm–Bonferroni procedure. The level of statistical significance was set at 0.05. All statistical analyses were performed using the JASP statistics package (version 0.18.3).

## 3. Results

Between-groups comparisons of baseline values for the different dependent variables are shown in Table 2. These results indicate that IOP was significantly lower, while SBP, BPP, and OPP were significantly higher in older adults compared to the young adult group. Additionally, VSMS-F and VSMS-B scores were significantly lower in the older group than in the young adult group.

These baseline differences indicate that the two groups differed in both ocular and cardiovascular profiles prior to task exposure. Therefore, subsequent analyses focused on within-subject changes across conditions and condition × group interactions to better capture task-related physiological responses beyond baseline variability.

**Figure 3:**
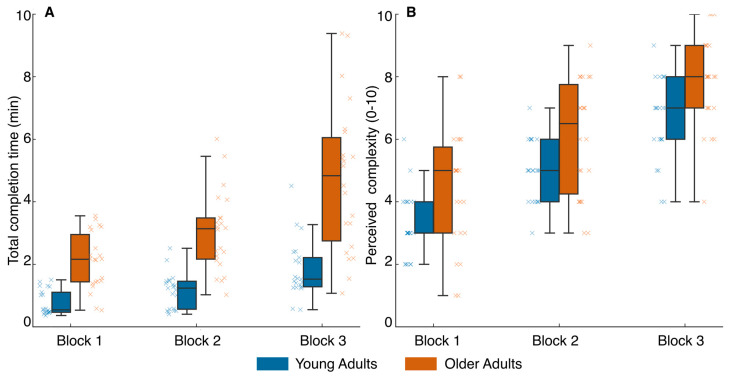
Task completion time (**A**) and perceived task complexity (**B**) across symbol identification blocks in young and older adults. Box plots display the median (central line), interquartile range (box), and range (whiskers). Individual data points are shown as overlaid “×” symbols. Blue = young adults; orange = older adults.

A two-way ANOVA applied to analyze the changes in the time required to complete the task revealed statistically significant differences for the main effects of task complexity (F_2,82_ = 44.8, *p* < 0.001, η^2^p = 0.52) and group (F_1,41_ = 42.5, *p* < 0.001, η^2^p = 0.51), as well as for the interaction task complexity × group (F_2,82_ = 8.1, *p* < 0.001, η^2^p = 0.17). Post hoc comparisons (Holm–Bonferroni corrected) showed significant differences between block 1 and block 2 (*p* = 0.005, Cohen’s *d* = 0.51), block 1 and block 3 (*p* < 0.001, Cohen’s *d* = 1.46), and block 2 and block 3 (*p* < 0.001, Cohen’s *d* = 0.95). Additionally, older adults required significantly more time than young adults across all blocks (*p* < 0.001, Cohen’s *d* = 1.60), with the largest between-group difference observed in block 3 (*p* < 0.001, Cohen’s *d* = 3.12). Task completion time increased progressively with task complexity, with a large effect size, and older adults performed consistently slower than young adults across all blocks. Importantly, the interaction effect indicates that these between-group differences became more pronounced as task difficulty increased (Figure 3A).

A two-way ANOVA revealed significant effects for perceived task complexity, including the main effects of task complexity (F_2,82_ = 122.1, *p* < 0.001, η^2^p = 0.75) and group (F_1,41_ = 8.9, *p* = 0.005, η^2^p = 0.18), whereas the interaction task complexity × group was not significant (F_2,82_ = 0.3, *p* = 0.868, η^2^p = 0.01). Post hoc comparisons showed significant differences between block 1 and block 2 (*p* < 0.001, Cohen’s *d* = 1.66), block 1 and block 3 (*p* < 0.001, Cohen’s *d* = 1.96), and block 2 and block 3 (*p* < 0.001, Cohen’s *d* = 1.24). Additionally, older adults reported significantly higher perceived task complexity than young adults across all blocks (*p* = 0.005, Cohen’s *d* = 0.91) (Figure 3B). This pattern suggests that while both groups perceived the increase in task demands similarly, older adults experienced a consistently higher subjective load.

**Figure 4:**
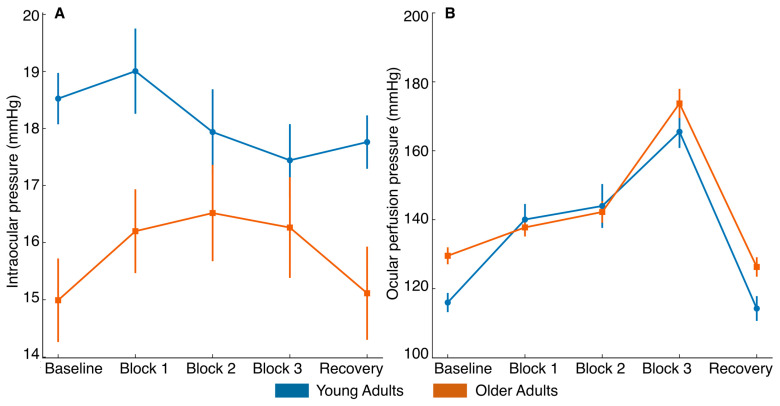
Intraocular pressure (**A**) and ocular perfusion pressure (**B**) across experimental phases. Mean intraocular pressure (mmHg) and ocular perfusion pressure (mmHg) were measured at baseline, during the three experimental blocks, and after recovery in young and older adults. Error bars represent standard error of the mean (SEM). Blue = young adults; orange = older adults.

A two-way ANOVA was applied for IOP, SBP, DBP, MAP, OPP, and RHR. Regarding IOP changes, statistically significant differences were found for the main effects of condition (F_4,164_ = 3.3, *p* = 0.012, η^2^p = 0.08) and group (F_1,41_ = 6.3, *p* = 0.016, η^2^p = 0.13) and the interaction condition × group (F_4,164_ = 4.0, *p* = 0.004, η^2^p = 0.09). Importantly, this interaction indicates that age differences were primarily reflected in the recovery phase rather than during task execution. Post hoc analysis revealed significant differences between block 1 and recovery (*p* = 0.011, Cohen’s *d* = 0.35). Specifically, young adults showed a reduction in IOP during recovery, whereas older adults exhibited a return toward baseline values, suggesting differences in short-term regulatory dynamics. Additionally, a between-group comparison showed a significant baseline difference in IOP (*p* = 0.001, Cohen’s *d* = 1.05) (Figure 4A).

**Figure 5:**
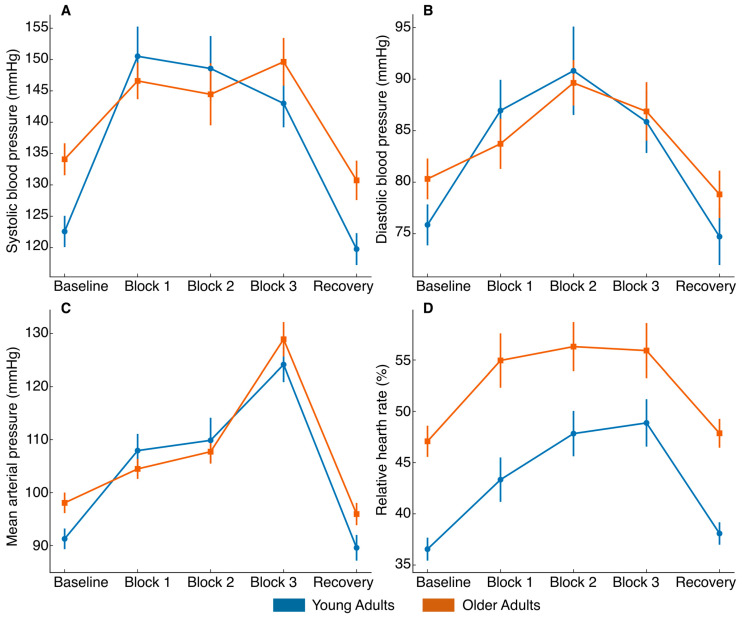
Cardiovascular responses across experimental phases. Systolic blood pressure (**A**), diastolic blood pressure (**B**), mean arterial pressure (**C**), and relative heart rate (**D**) were measured at baseline, during the three task blocks, and after recovery in young and older adults. Data are presented as group means ± standard error of the mean (SEM). Blue = young adults; orange = older adults.

For vascular factors, significant differences in OPP were found for the effect of condition (F_4,164_ = 129.1, *p* < 0.001, η^2^p = 0.76) and the condition × group interaction (F_4,164_ = 4.7, *p* = 0.002, η^2^p = 0.10), though no significant between-group differences were detected (F_1,41_ = 1.8, *p* = 0.193, η^2^p = 0.04). Similarly, for SBP, significant effects were observed for condition (F_4,164_ = 42.0, *p* < 0.001, η^2^p = 0.51) and condition × group (F_4,164_ = 5.0, *p* = 0.003, η^2^p = 0.11), but not for group (F_1,41_ = 1.0, *p* = 0.331, η^2^p = 0.02). For DBP, only the main effect of condition was statistically significant (F_4,164_ = 18.6, *p* < 0.001, η^2^p = 0.31). Lastly, for MAP, significant effects were observed for condition (F_4,164_ = 128.7, *p* < 0.001, η^2^p = 0.76) and condition × group (F_4,164_ = 4.2, *p* = 0.004, η^2^p = 0.09), with no between-group differences (F_1,41_ = 0.6, *p* = 0.450, η^2^p = 0.01). Post hoc analysis revealed a linear increase in OPP, SBP, DBP, and MAP throughout the tasks, followed by a return to baseline levels after five minutes of recovery (Figure 4B and Figure 5A–C). Overall, these results indicate a consistent physiological response to increasing task demands, characterized by progressive cardiovascular activation during the tasks and a return toward baseline during recovery.

For RHR, a statistically significant effect was obtained for the main effect of condition (F_4,164_ = 27.39, *p* < 0.001, η^2^p = 0.40) and group (F_1,41_ = 16.40, *p* < 0.001, η^2^p = 0.29), with older adults showing consistently higher RHR values than young adults. However, the condition × group interaction did not exhibit statistical significance (F_4,164_ = 0.87, *p* = 0.484, η^2^p = 0.02). Post hoc comparisons revealed significant between-group differences at baseline (*p* < 0.001, Cohen’s *d* = 1.11), block 1 (*p* = 0.016, Cohen’s *d* = 0.71), block 2 (*p* < 0.001, Cohen’s *d* = 1.19), block 3 (*p* < 0.001, Cohen’s *d* = 1.30), and recovery (*p* < 0.001, Cohen’s d = 1.19), confirming a consistently elevated RHR in the older group across all time points (Figure 5D). Overall, these results indicate that RHR increased across the protocol and remained consistently higher in older adults, while the pattern of change over time was similar in both groups.

## 4. Discussion

The present study examined age-related differences in ocular and cardiovascular responses to graded increases in motor–cognitive task complexity. The main findings were that increasing task complexity was associated with longer task completion time, higher perceived task complexity, and progressive changes in ocular and cardiovascular measures across the protocol. Age-related differences were most evident in task performance, consistently higher RHR in older adults, and in the temporal pattern of IOP, OPP, SBP, and MAP responses, with the clearest difference observed for IOP during early recovery.

With respect to behavioral outcomes, increasing task complexity led to longer completion times, and older adults performed more slowly across all blocks. Older adults also gave higher complexity ratings than young adults, although both groups showed a comparable increase in these ratings as task difficulty increased. This pattern suggests that, in older adults, cognitively demanding tasks may be experienced as more subjectively burdensome, which may contribute to slower performance as task complexity increases [28].

Together with the lower visuospatial memory scores observed in older adults, their slower task performance across successive blocks may reflect a greater impact of increasing task complexity on working-memory resources. This interpretation is broadly consistent with evidence showing that, with advancing age, performance in dual-task situations becomes more dependent on cognitive function and more vulnerable to increasing task complexity [7,11]. Previous studies have also reported greater dual-task interference in older adults, particularly when cognitive processing must be combined with postural or locomotor control [29,30]. It should be noted, however, that the extent of interference in dual-task situations may also depend on task characteristics and on the strategy adopted during task performance. For example, participants may prioritize the motor component, prioritize the cognitive component, or attempt to maintain both at a comparable level [31,32]. Overall, these considerations help to contextualize the age-related differences in task performance observed across the protocol.

Among the physiological measures, the clearest age-related differentiation was observed for IOP, particularly at the recovery assessment. In young adults, IOP showed a more pronounced decrease from block 1 to recovery, whereas in older adults it returned toward baseline values, suggesting age-related differences in the short-term pattern of response. The pattern observed in young adults may be related to previous findings from studies of high-intensity aerobic exercise. Alfaqeeh et al. [33], who assessed IOP before, during, and after aerobic exercise performed at 50%, 70%, and 85% of peak power in healthy young men, reported a significant reduction in IOP under the highest-intensity condition. Thus, the more pronounced decrease in IOP observed in our young adults may reflect a pattern similar to that reported after high-intensity dynamic exercise, although those studies examined isolated aerobic exercise rather than combined motor–cognitive load. In older adults, the less pronounced decline in IOP and the return toward baseline at recovery suggest that the IOP response differed from that observed in young adults, particularly in its temporal profile. This interpretation is broadly consistent with previous studies reporting age-related differences in ocular parameters, including IOP, as well as age-related variation in the temporal profile of IOP changes [34,35,36]. Taken together, these findings indicate that age-related differences in IOP were reflected not only in baseline level but also in the time course of post-task recovery.

Cardiovascular measures showed progressive increases across the task blocks, followed by a reduction at recovery, indicating that the graded motor–cognitive protocol elicited a progressively stronger hemodynamic response in both groups. In addition, older adults showed consistently higher RHR values across the protocol, which should be interpreted as a greater cardiovascular load relative to age-estimated capacity rather than simply as a higher absolute HR response. The stepwise increase in SBP across blocks suggests that each successive stage of the protocol imposed greater task complexity and required greater cardiovascular adjustment during performance. This interpretation is supported by previous work treating SBP as a useful index of cognitive effort and task engagement [14], as well as by models proposing that effort mobilization increases with task difficulty [12]. It is also broadly consistent with age-comparative evidence showing that SBP responses track engagement during cognitively demanding activity and may be particularly informative in older adults [28]. At the same time, the age effect observed in the present study was not expressed as consistently higher SBP or MAP levels in older adults, since no significant main effect of group was found for either variable. Instead, age-related differences were reflected in the way SBP and MAP changed across the protocol, particularly during the early recovery assessment. Thus, the significant condition × group interactions, together with their small-to-moderate effect sizes, indicate modest differences in the temporal profile of the response rather than pronounced cardiovascular separation between the groups. Taken together with the consistently higher RHR, this pattern suggests that older adults performed the protocol under a greater relative cardiovascular load, which may be relevant when considering task progression and physiological monitoring across age groups. This interpretation should be viewed cautiously and considered in context, but it is broadly consistent with observations suggesting that age may influence the temporal profile of cardiovascular responses to acute task-related load [37]. By contrast, DBP showed a general condition effect without age-related differentiation.

OPP is best interpreted as an outcome index reflecting concurrent changes in arterial blood pressure and IOP [38]. Therefore, its increase across the task blocks and return toward baseline at recovery should be considered in relation to both systemic and ocular responses to increasing motor–cognitive load. Although no significant main effect of group was found for OPP, the significant condition × group interaction indicates a modest age-related difference in the pattern of change across the protocol. Thus, OPP appears to reflect a strong condition effect, accompanied by limited age-related differentiation in response profile.

A key contribution of the present study is the integrated characterization of ocular and cardiovascular responses across successive levels of motor–cognitive task complexity and during early recovery in young adults and older adults. While earlier studies have typically examined ocular and cardiovascular responses separately, either in exercise-related or cognitively demanding contexts [8,13,15,16], the present approach made it possible to capture changes in both systems within a single graded motor–cognitive protocol. This is particularly relevant because ocular responses, especially patterns of IOP change, are known to depend on multiple interacting factors, including exercise modality, intensity, body position, breathing pattern, fitness level, and mental effort [17,18]. Against this background, the present findings suggest that graded motor–cognitive load and age may influence the temporal pattern of ocular responses, particularly IOP.

From a practical perspective, the combined assessment of ocular and cardiovascular parameters may provide a more comprehensive framework for understanding how healthy adults adapt to complex motor–cognitive demands. This may be especially relevant in the context of healthy aging, as cognitive–motor dual-task performance is closely related to everyday functional demands [10]. In applied settings, these findings may provide a useful reference for the design and structuring of motor–cognitive exercise formats, including task-based systems such as SKILLCOURT. They also suggest that both the progression of task complexity and the early recovery response should be taken into account when planning such activities in healthy adults across age groups.

Several limitations should be acknowledged. First, recovery was assessed at a single post-task time point (5 min after task completion), which limits a more detailed characterization of the temporal dynamics of physiological recovery. Although this time window has previously been shown to be sufficient to capture short-term intraocular pressure recovery following physical exertion [39], it does not allow a more detailed analysis of recovery kinetics or trajectory over time. Therefore, the present findings should be interpreted as reflecting early post-task adjustment rather than complete recovery dynamics. Second, the relatively small sample and the inclusion of only healthy participants limit the generalizability of the findings to clinical or at-risk populations, including individuals with glaucoma, cardiovascular conditions, or lower functional capacity. Although the sample size was supported by an a priori power analysis, the study was primarily powered to detect within-subject effects rather than complex interaction effects with the same precision. Accordingly, the observed condition × group interactions should be interpreted cautiously and confirmed in larger samples. Third, habitual physical activity was not formally assessed, and no objective measure of physical fitness was obtained in either group. Therefore, the observed physiological variability may have reflected not only age-related differences but also variation in physical activity status. Fourth, behavioral performance during the graded motor–cognitive protocol was assessed primarily by task completion time, which did not allow a direct evaluation of response accuracy or potential speed–accuracy trade-offs. Finally, the physiological protocol was based on standard cardiovascular and ocular measures and did not include additional autonomic indices. In addition, blood pressure was measured with a wrist device under standardized conditions, although wrist-based measurements may be somewhat more susceptible to positioning-related error than validated upper-arm monitoring approaches [40]. Future studies should incorporate multiple post-task assessments to better characterize recovery processes, together with more detailed autonomic measures, alternative blood pressure monitoring methods, and broader assessment of individual fitness-related factors.

## 5. Conclusions

Graded motor–cognitive task complexity elicited progressive ocular and cardiovascular responses in both groups. Age-related differences were most evident in task performance, the consistently higher RHR observed in older adults, and selected physiological responses, with the clearest difference observed for IOP during early recovery. The concurrent assessment of ocular and cardiovascular measures within a single graded motor–cognitive protocol extends the interpretation of age-related differences beyond behavioral outcomes alone. These findings may provide a useful basis for future studies using a similar approach in larger samples and, in the longer term, in clinical or at-risk populations.

## Figures and Tables

**Table 1:** Demographic and baseline characteristics of the study participants.

	Young Adults			Older Adults		
	All	Women	Men	All	Women	Men
Sample size (n)	21	10	11	22	13	9
Age (years)	27.1 ± 7.7	28.6 ± 8.4	25.8 ± 7.0	63.1 ± 7.7	63.1 ± 7.2	63.2 ± 9.0
Body mass (kg)	73.1 ± 15.5	63.1 ± 7.8	82.3 ± 15.3	72.2 ± 14.3	62.8 ± 6.2	85.9 ± 11.3
Body height (cm)	174.4 ± 11.7	165.9 ± 4.5	182.1 ± 10.8	169.4 ± 11.3	162.6 ± 7.5	179.2 ± 8.1
BMI (kg/m^2^)	23.9 ± 3.5	22.9 ± 2.3	24.8 ± 4.2	25.0 ± 3.1	23.8 ± 2.9	26.7 ± 2.6

Note: Values are presented as mean ± standard deviation, BMI—Body Mass Index.

**Table 2:** Baseline comparisons of intraocular pressure, cardiovascular parameters, and visual–spatial memory between age groups.

Sample Characteristics	Young Adults	Older Adults	*p*-Value (ES)
IOP (mmHg)	18.5 ± 2.1	15.0 ± 3.4	0.002 (0.56, large) *
SBP (mmHg)	122.6 ± 11.4	134.1 ± 12.0	0.002 (0.98, large)
DBP (mmHg)	75.9 ± 9.1	80.3 ± 9.3	0.119 (0.49, small)
BPP (mmHg)	46.7 ± 9.3	53.8 ± 10.0	0.021 (0.73, medium)
HR (bpm)	70.3 ± 8.6	73.6 ± 10.4	0.258 (0.35, small)
OPP (mmHg)	116.0 ± 12.7	130.0 ± 11.5	<0.001 (1.12, large)
VSMS-F (n)	4.6 ± 0.7	3.5 ± 1.0	<0.001 (1.25, large)
VSMS-B (n)	4.6 ± 0.9	3.2 ± 0.9	<0.001 (1.4, large)

Note: IOP—intraocular pressure; SBP—systolic blood pressure; DBP—diastolic blood pressure; BPP—blood pulse pressure; HR—heart rate; OPP—ocular perfusion pressure; VSMS-F—visual–spatial memory span test, forward version; VSMS-B—visual–spatial memory span test, backward version; * Mann–Whitney; ES—effect size; ES is interpreted as small, medium, and large according to Cohen [27] thresholds (d: 0.2, 0.5, and 0.8, respectively). For the Mann–Whitney U test, ES was reported as r (0.1 small, 0.3 medium, 0.5 large).

## Data Availability

The data presented in this study are available on request from the corresponding author due to ethical and privacy restrictions related to human participant data and the need to protect participant confidentiality.

## Abbreviations

The following abbreviations are used in this manuscript:

BP	Blood pressure
BPP	Blood pulse pressure
DBP	Diastolic blood pressure
HR	Heart rate
IOP	Intraocular pressure
MAP	Mean arterial pressure
OPP	Ocular perfusion pressure
RHR	Relative heart rate
SBP	Systolic blood pressure
VSMS-B	Visual–spatial memory span test, backward version
VSMS-F	Visual–spatial memory span test, forward version
